# The oldest continuous association between astigmatid mites and termites preserved in Cretaceous amber reveals the evolutionary significance of phoresy

**DOI:** 10.1186/s12862-025-02351-5

**Published:** 2025-02-25

**Authors:** Hemen Sendi, Pavel B. Klimov, Vasiliy B. Kolesnikov, Júlia Káčerová, Enrico Bonino, Dany Azar, Ninon Robin

**Affiliations:** 1 Institute of Zoology, Slovak Academy of Sciences, Dúbravská cesta 9, Bratislava, 84506 Slovakia; 2 Dpt of Biological Sciences, Purdue University, 915 Mitch Daniels Blvd, West Lafayette, IN USA; 3 X-BIO Institute, Tyumen State University, Tyumen, 625003 Russia; 4 Papanin Institute for Biology of Inland Waters, Russian Academy of Sciences, Borok, Yaroslavl Province 152742 Russia; 5 Department of Sculpture, Object and Installation, Academy of Fine Arts and Design, Hviezdoslavovo nám. 18, 814 37, Bratislava, Slovakia; 6 Rue G. Thiriart 74, Liège, 4000 Belgium; 7 Nanjing Institute of Geology and Palaeontology, Chinese Academy of Sciences, 39 East Beijing road, Nanjing, 210008 China; 8 Faculty of Science II, Natural Sciences Department, Lebanese University, Fanar, Matn 90656 Lebanon; 9 Géosciences Rennes – UMR 6118, Univ Rennes, CNRS, 263 Général Leclerc avenue, Rennes, 35042 France; 10 Directorate Earth and History of Life, Royal Belgian Institute of Natural Sciences, 29 Vautier Street, Brussels, 1000 Belgium

**Keywords:** Isoptera, Lower Cretaceous, Barremian, Social insects, Lebanese amber

## Abstract

**Background:**

Among minute-sized and wingless arthropods, astigmatid mites stand out for their diverse range of symbiotic associations (parasitic, neutral and mutualistic), with both invertebrate and vertebrate hosts. When inhabiting discontinuous and ephemeral environments, astigmatid mites adapt their life cycle to produce a phoretic heteromorphic nymph. When feeding resources are depleted, phoretic nymphs disperse to new habitats through phoresy, attaching to a larger animal which transports them to new locations. This dispersal strategy is crucial for accessing patchy resources, otherwise beyond the reach of these minute arthropods. In Astigmata, the phoretic nymph is highly specialized for dispersal, equipped with an attachment organ and lacking a mouth and pharynx. Despite the common occurrence of phoretic associations in modern mites, their evolutionary origins remain poorly understood. Among Astigmata, the family Schizoglyphidae represents an early derivative lineage with phoretic tritonymphs; however, our knowledge of this family is limited to a single observation.

**Results:**

Here, we report the oldest biotic association of arthropods fossilised in amber (~ 130 Ma, Lebanon): an alate termite with 16 phoretic tritonymphs of Schizoglyphidae (*Plesioglyphus lebanotermi* gen. et sp. n.). The mites are primarily attached to the membranes of the host’s hindwings, using their attachment organs, pretarsal claws and tarsal setae. Additionally, we report new modern phoretic tritonymphs of this same family, on one of the earliest lineages of termites. These data collectively indicate that schizoglyphid-termite associations represent the oldest continuous mite-host associations. Notably, phoretic schizoglyphids retain a distinct mouth and pharynx, whereas these structures are absent in the modern phoretic stages of non-schizoglyphid Astigmata.

**Conclusion:**

The discovery of Schizoglyphidae mites in Lebanese amber represents the oldest known continuous association between acariform mites and their hosts. This finding demonstrates the long-term evolutionary significance of phoresy in Astigmata, evidencing a relationship sustained for over 130 Ma. It indicates that these early mites lived inside termite nests as inquilines and used alate termites for dispersal. This ancient association offers key insights into the coevolution of both mites and termites, highlighting a potential for the future discoveries of similar mites. This fossil —a stem-group Astigmata— is important for the accurate calibration of acariform mite phylogenies, advancing our understanding of these mites evolutionary history.

**Supplementary Information:**

The online version contains supplementary material available at 10.1186/s12862-025-02351-5.

## Background

Phoresy is a symbiotic interaction in which one life stage of a smaller animal attaches to a larger animal to facilitate dispersal [1–2] and access a more favourable habitat [3]. While reflecting primarily commensalism [4], phoresy can be defined together with other types of symbiotic interactions like mutualism [5], parasitism [6], or parastioidism [1, 7] as part of the lifestyle of the phoretic organism. For relatively small and/or wingless arthropods (springtails, pseudoscorpions and mites), phoresy is a key strategy to provide access to distant and patchy resources beyond their normal reach [8–9]. Phoretic organisms exhibit host-seeking behaviours and various adaptations for attachment; they typically do not feed, nor reproduce, during transport on their hosts [1].

Mites (Acari), with around 55,000 described species, are among the most diverse arthropods, representing a clade of substantial medical and economical importance. They exhibit a range of ecological lifestyles, including free-living, parasitism, active predation, and saprophagy, particularly in soil-dwelling mites [10–12]. Modern mites, although they may not form a monophyletic group, include two monophyletic superorders: Acariformes (32,000 species) and Parasitiformes (23,000 species) [13]. Phoresy has evolved and disappeared multiple times throughout mite evolution [1]. Among the Acariformes, phoresy is common in Heterostigmata (2,700 species) and Astigmata (6,300 species) [1]. In Heterostigmata, females are typically the dispersal stage, whereas in most free-living Astigmata, the heteromorphic nymph—formerly known as the ‘hypopus’—serves as the dispersal stage [14–15]. In the enigmatic astigmatid family Schizoglyphidae, the dispersal stage is likely a tritonymph [16], which morphologically resembles the heteromorphic deutonymph. Astigmatid heteromorphic nymphs possess a highly specialized attachment organ, featuring various suckers and adhesive conoids adapted to different attachment sites, such as smooth insect cuticle, setae, or mammal hair [15]. Astigmata have a shorter life cycle compared to their ancestors, oribatid mites. While oribatid mites inhabit soil—an uninterrupted habitat—Astigmata prefer discontinuous and ephemeral environments, such as decomposing plant and fungal materials, stored products, phytotelmata, dung, actively growing mycelia, subcortical spaces, tree sap flows, and invertebrate and vertebrate nests. This habitat preference makes long-distance dispersal a crucial component of their life cycle, and Astigmata establish phoretic associations with larger organisms, such as mammals, insects, and myriapods [14]. Astigmatid phoretic nymphs often travel in groups to enhance sexual reproduction at their destination and increase their chances of establishing a large population on new resources, thereby outcompeting other colonizers [1]. Unlike other life stages (larvae, non-phoretic nymphs, and adults), phoretic nymphs are typically non-feeding, although there are occasional exceptions [6]. Adults are the sole reproductive stage. A few free-living Astigmata are capable of dispersing as adults [1, 14].

Phoresy is likely the ancestral lifestyle of Astigmata, but it has been lost in (i) astigmatids inhabiting continuous habitats (such as soil or water), (ii) associated with non-nest-building insects; or (iii) nearly all vertebrates with overlapping generations, which enables maternal vertical transmission [1, 14]. Astigmata have experienced three major evolutionary events: (1) the ancestral oribatid life cycle (lacking a phoretic deutonymph) evolved to a life cycle with a specialized phoretic deutonymph (non-schizoglyphid Astigmata) or tritonymph (schizoglyphid Astigmata); (2) the deutonymphal lifecycle was then modified to permanently suppress the deutonymphal stage in several lineages, notably in Psoroptidia, a lineage of full-time (permanent) associates of birds and mammals, as these mites could effectively colonize new hosts via vertical transmission and other direct host-to-host contacts; (3) Pyroglyphidae, a lineage within Psoroptidia, transitioned to living in the nests of their hosts, thereby becoming secondarily free-living and using their former hosts for transport without forming phoretic heteromorphic nymphs [1, 17–19].

The family Schizoglyphidae is the sister group to the remaining extant Astigmata [1]. This family is distinctive for retaining several plesiomorphic traits, including the relative position of the genital opening and attachment organ, as well as the structure of the attachment organ and gnathosoma compared to other astigmatid families [20]. This monotypic family includes a single observation of two specimens of the extant *Schizoglyphus biroi* Mahunka, 1978, found in New Guinea (Indonesia) on a tenebrionid beetle. Although this species exhibits the specific morphological adaptations characteristic of a phoretic heteromorphic deutonymph, it is probably a tritonymph [16]. Evidence for this includes the presence of three pairs of genital papillae (as seen in oribatid tritonymphs and adults) instead of the two pairs found in deutonymphs and adults of other Astigmata. Despite substantial efforts to find this mite again on tenebrionid beetles, it has not been re-collected, suggesting that the original phoretic host (the beetle) may represent an incidental record rather than a true biologically relevant association. Thus, the true association of *Schizoglyphus biroi* and the family it represents remains elusive.

Acariformes is one of the earliest diverging groups within the arachnids, with fossil evidence dating back to the early Devonian [21]. However, symbiotic associations with other organisms are mostly documented from the Cretaceous, when amber preserved these interactions in situ [14, 22–23]. Crown-group Astigmata are estimated to have diverged between the Late Permian and the Early Triassic, while the stem-group likely originated in the Late Devonian to Carboniferous [13–14, 17, 22–23]. In the fossil record, Astigmata appears from the Cretaceous onward, becoming more abundant in the Cenozoic, with several instances of biotic associations [24–32].

**Fig. 1:**
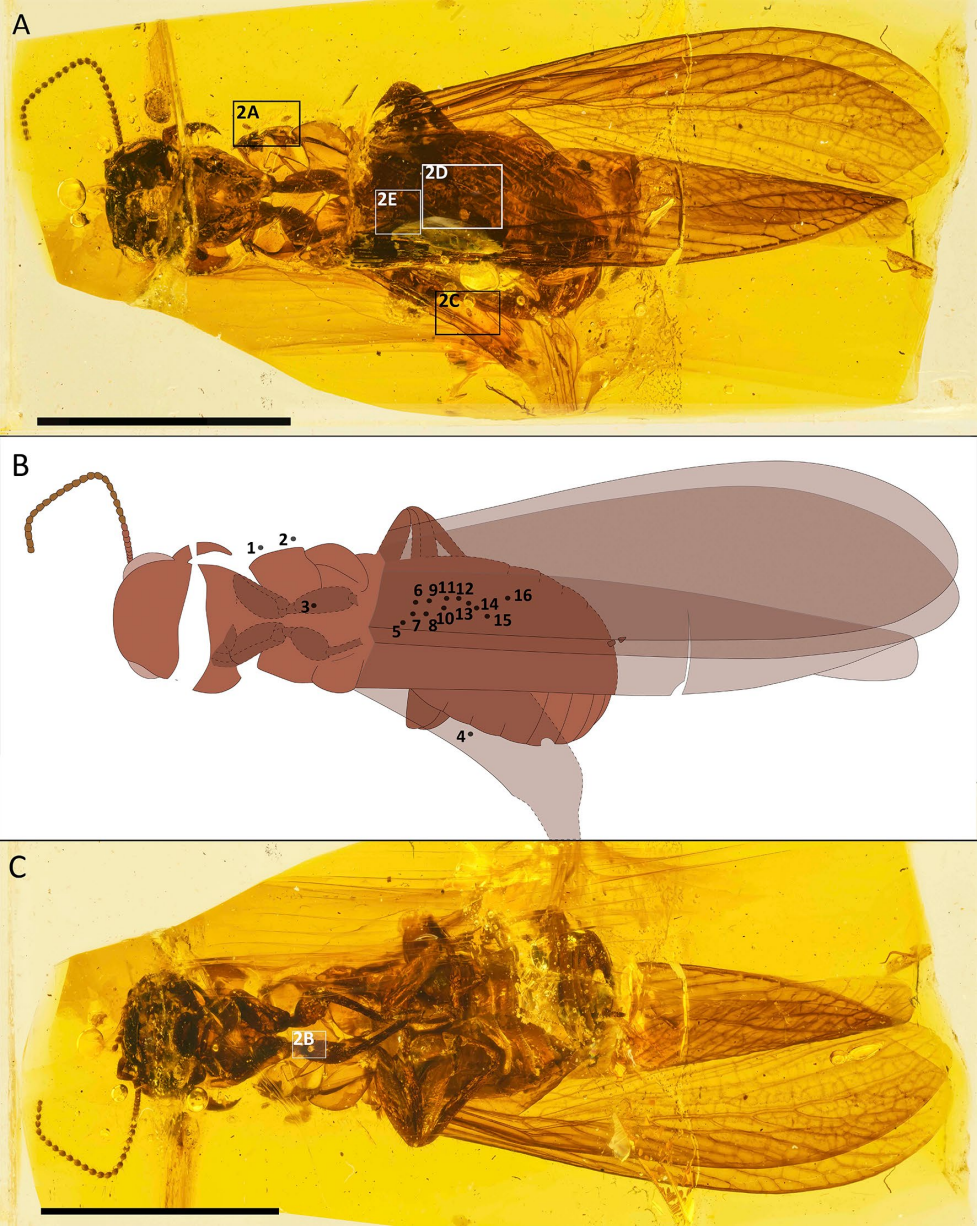
Overview of the termite *Lebanotermes veltzae* Engel, Azar et Nel, 2011 from Barremian Lebanese amber with localization of phoretic schizoglyphid mites. (**A**) Dorsal view of the termite with four highlighted areas indicating the occurrence of the mite *Plesioglyphus lebanotermi* gen. et sp. n. (**B**) Mite localization map. Mites are positioned as follows: 1, 4, 5, 8, 12, 15 ventral; 10, 11, 16 dorsal; 2, 6, 7, 9, 13, 14 dorsolateral; 3 frontal. (**C**) Ventral view of termite with highlighted occurrence of a mite on the host foreleg. Boxes refer to detailed photographs in Fig. 2. Scale bars: 5 mm

Here, we report the oldest amber association of an phoretic arthropod associated with its host preserved in Lebanese amber (Early Barremian, ~ 130 Ma): 16 mite tritonymphs belonging to the family Schizoglyphidae, phoretic on an alate termite *Lebanotermes veltzae* Engel, Azar et Nel, 2011 (Figs. 1; and 2). To confirm this association and explore potential true hosts of *Schizoglyphus biroi*, we also examined nests of modern termites in New Zealand and found schizoglyphids associated with *Stolotermes*, one of the earliest diverging termite lineages. The combined fossil and modern evidence suggests that schizoglyphid-termite associations represent the oldest known ongoing relationship between mites and their hosts. These findings shed light on the range of plesiomorphic features (including the gnathosoma and the attachment organ), present in the earliest crown-group Astigmata and reveal a highly conserved phoretic morphology that has persisted since the Early Cretaceous. This also advances our understanding of the temporal framework for the diverse mite-arthropod ecological associations observed today.

## Methods

We studied 16 mite specimens attached to the fossil holotype of the termite *Lebanotermes veltzae* described in Engel et al. [33]. The termite host was preserved in a single amber piece (341 C–T) from the Lower Barremian of Mdeyrij-Hammana, Caza (District) Baabda, Lebanon, coll. D. Azar. The amber piece is housed at the Natural History Museum of the Lebanese University, Faculty of Sciences II, Fanar. Other syninclusions derived originally from the same amber block include: the allotype of the chironomid dipteran *Ziadeus kamili* (341 B), an aleyrodid hemipteran (341 A), and a ceratopogonid dipteran. The amber piece was trimmed and polished for microscopy, embedded in Canada balsam and placed in a cube of 1 mm thick microscopic glass slides. This permanent glass-amber preparation makes computed tomography not feasible for resolutions targeting the 150 μm-long mites.

**Fig. 2:**
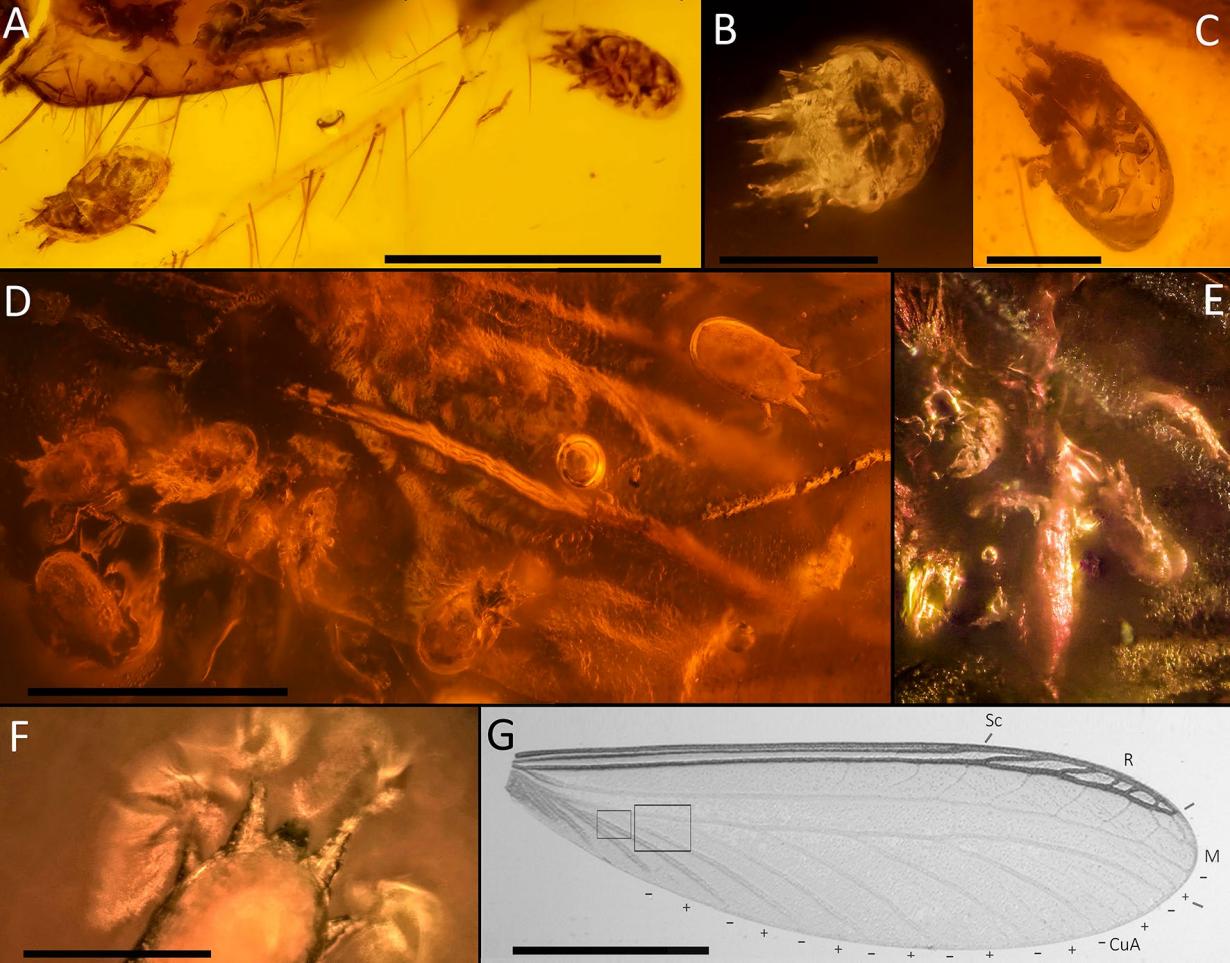
Attachment of the schizoglyphid mite *Plesioglyphus lebanotermi* gen. et sp. n. on the termite from Lebanese amber, Barremian. (**A**) Specimens 1 and 2 detached ventrolaterally from the termite host. (**B**) Specimen 3 is attached to the forefemur of the termite host. (**C**) Specimen 4 is attached between the hindwings of the host. (**D**) Specimens 9–15 are attached between the termite hindwings above the dorsal abdominal area. (**E**) Specimens 5–8 are attached between the termite hindwings above the dorsal abdominal area. (**F**) Detail of attachment of specimen 16 on the wing membrane of its host. (**G**) – +/- schematics of the CuA area of an extant termite hindwing adapted from Scheffrahn et al. [20] (CC BY 4.0) similar to the area with attached mites in *Lebanotermes veltzae* Engel, Azar et Nel, 2011. Note that the wings of termites are not as corrugated as in Paleozoic insects or in Odonatoptera, and Blattodea. Scale bars: 500 μm (**A**, **D**), 100 μm (**B**–**C**), 0,3 mm (**E**), 150 μm (**F**), 2 mm (**G**)

Several microphotographs (Figs. 1A, C; 2A–D, F; and 4A–C) were acquired using the protocol described on this website (https://enrico-bonino.eu): a Sony a7R II mirrorless camera with a 208 mm tube lens, a Raynox DCR-150, microscope objectives Mitutoyo QV 2.5× (or APO 20x), and a 110 mm tube lens with inversed Schneider Componon-S 50 mm/f2.8 lens for the whole specimen. This system (camera, tube-lens, and optics) is mounted on a MJKZZ Ultra Rail MINI V2, allowing movements in both vertical and horizontal planes. The illumination was provided by a cylindrical OGGLAB LED system DB 120EB. The entire panoramic image was assembled from two overlapping stacks, each composed of 53 frames. Images were captured in 16-bit RAW format with several steps between frames: 15 μm (with the Mitutoyo QV 2.5x), 2 μm (with the Mitutoyo 20x), and 95 μm (with Schneider-Componon lens). The frames were sub-stacked per group of eight images, stacked secondarily together and retouched in Helicon Focus (v.8.2, Professional) to remove undesired features (e.g. bubbles, dust, surfaces not in focus). Final image post-processing was done with Adobe Photoshop and Topaz DeNoise software. We also used a Zeiss Axiocam 208 mounted on an Axioimager A2 microscope, equipped with EC Epiplan 20x/0.4, 50x/0.55, and a W N-Achroplan 63 × 0.9 (water immersion) both in reflection and transmission light mode.

Multispectral microimaging was performed at a research platform IPANEMA (SOLEIL Synchrotron, Orsay, France, microscope magnification x20). Reflection and luminescence images emitted by the sample were collected in various spectral ranges using a setting coupling (1) an illumination device employing 16 different LED lights (from 365 up to 700 nm wavelength, CoolLED pE-4000) and (2) a light filter device placed in front of the camera detector (a wheel holding six interference band-pass filters collecting signal within in six spectral ranges from 435 to 935 nm). Out of the 96 produced illumination/detection couples, a selection of three couples enhancing morphological features of interest were combined into pseudo-colored RGB. The stacking, alignment, image registration of the different couples, and production of pseudo-colored RGB composites were performed using ImageJ. Pseudo-colored RGB images were produced with red—illumination 435 nm/detection 650 ± 60 nm (luminescence), green—470 nm/det. 650 ± 60 nm, blue—470 nm/det. 732 ± 68 nm.

## Results

### Systematic paleontology

Class **Arachnida** Cuvier, 1812.

Superorder **Acariformes** Zachvatkin, 1952.

Order **Sarcoptiformes** Reuter, 1909.

Suborder **Oribatida** Dugès, 1834.

Hyporder **Astigmata** Canestrini, 1891.

Family **Schizoglyphidae** Mahunka, 1978 (type genus *Schizoglyphus* Mahunka, 1978).

***Plesioglyphus*** **gen. n.**

urn: lsid: zoobank.org: act:7BF9A0B3-8682-4E9A-95FB-8BCB265C43E3.

**Type species.***Plesioglyphus lebanotermi* gen. et sp. n.

**Fig. 3:**
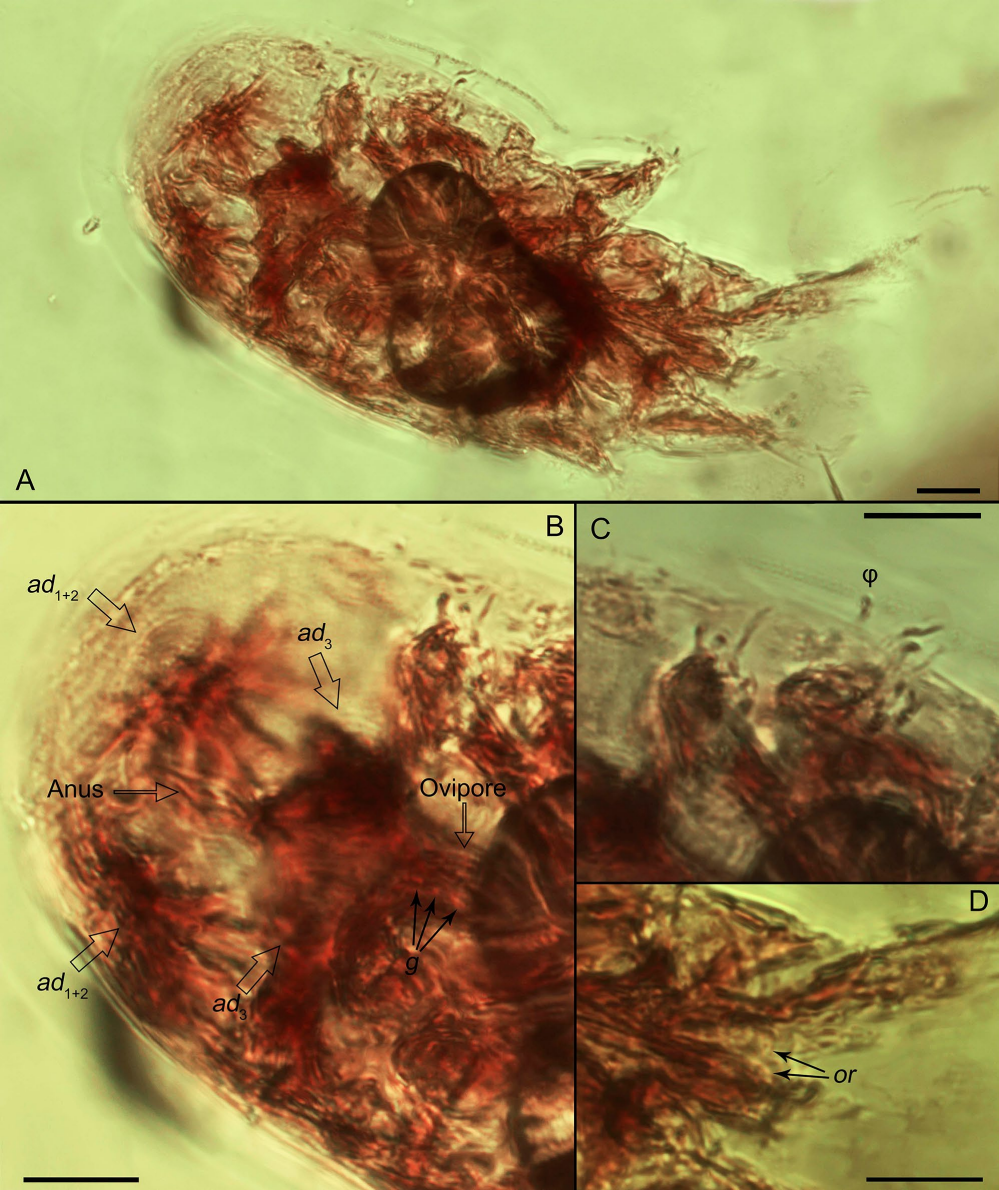
Light microscope images of the schizoglyphid tritonymph of *Plesioglyphus lebanotermi* gen. et sp. n., holotype (341 C), Lower Barremian Lebanese amber of Mdeyrij-Hammana, ventral view (**A**) Total view. (**B**) Posterior part. (**C**) Left legs III and IV. (**D**) Anterior part and gnathosoma. Abbreviations: *ad –* suckers; φ *–* tibial solenidion; *g –* genital setae; *or–*adoral setae. Scale bars 20 μm

**Type material.** Holotype (341E): phoretic tritonymph, specimen 1 (Figs. 2A; 3; and 5C), attached to alate specimen of the termite *Lebanotermes veltzae* (holotype), embedded in single amber piece (341 C–T), the Lower Barremian of Mdeyrij-Hammana, Caza Baabda, Lebanon, coll. D. Azar, preserved at the Natural History Museum of the Lebanese University, Faculty of Sciences II, Fanar. Paratypes: 16 phoretic tritonymphs (341 F–T), same data.

**Type locality and horizon.** Mdeyrij-Hammana, Caza (District) Baabda, Mount Lebanon Governorate, Lebanon, Lower Cretaceous, Lower Barremian.

**Fig. 4:**
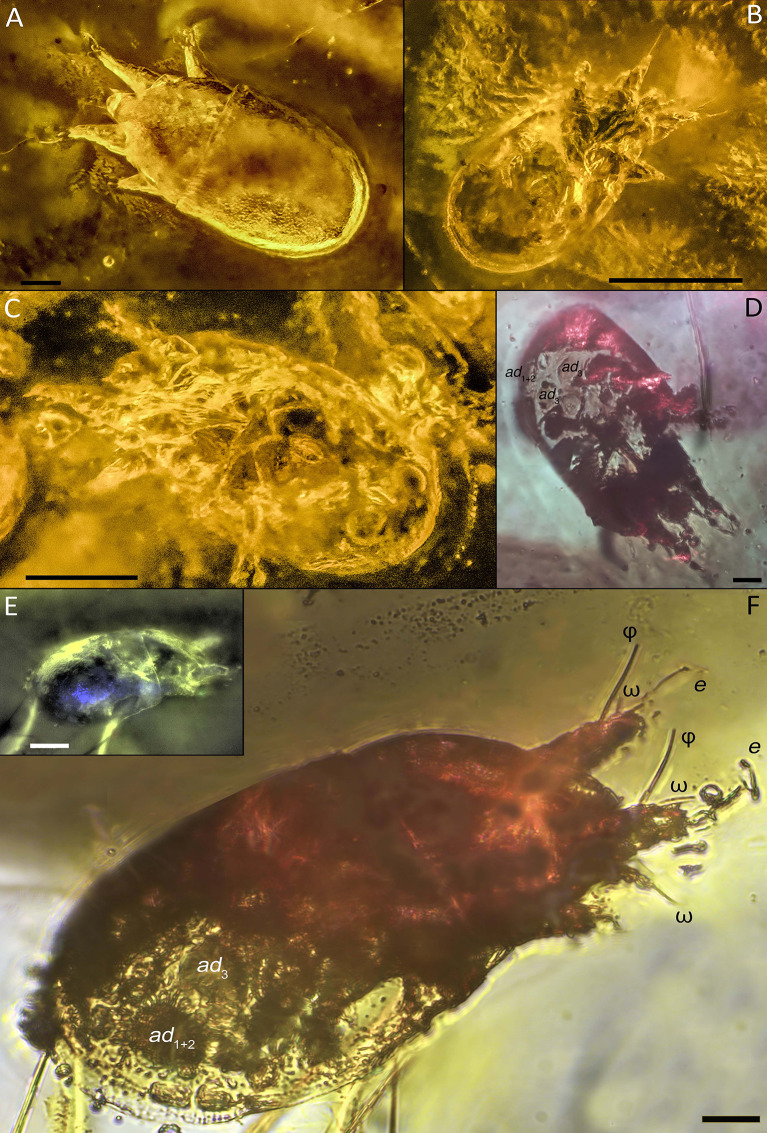
Light microscope images of the schizoglyphid tritonymph *Plesioglyphus lebanotermi* gen. et sp. n. from Lower Barremian Lebanese amber of Mdeyrij-Hammana (**A**) Dorsal view of specimen 16. (**B**) Ventral view of specimen 15. (**C**) Ventral view of specimen 12, paratype. (**D**) Ventral view of specimen 4. (**E**-**F**) Dorso-lateral view of specimen 2. Abbreviations: *ad –* suckers; ω * –* palp solenidion; φ * –* tibial solenidion; *e –* tarsal I seta. Scale bars 20 μm (**A**, D –**F**), 120 μm (**B**), 60 μm (**C**)

**Fig. 5:**
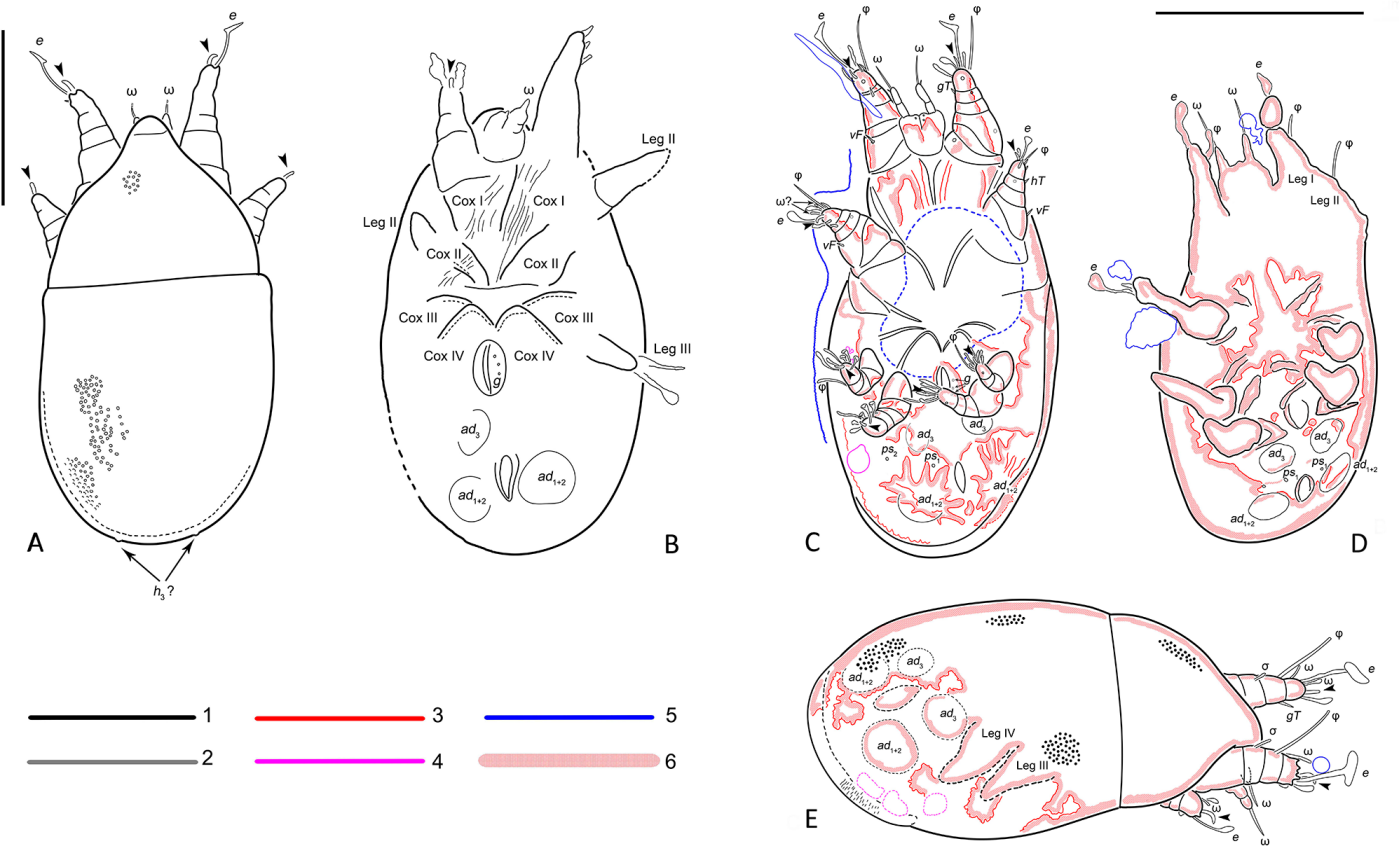
Line drawings of the schizoglyphid tritonymph *Plesioglyphus lebanotermi* gen. et sp. n. from Lower Barremian Lebanese amber of Mdeyrij-Hammana (**A**) Dorsal view of specimen 16. (**B**) Ventral side of specimen 15. (**C**) Ventral side of specimen 1, holotype. (**D**) Ventral side of specimen 4. (**E**) Dorso-lateral side of specimen 2. **(F)** Color codes for C-E: 1 – mite, 2 – presumably mite, 3 – mite internal structure, 4 – unknown structures, 5 – artifacts and sub-inclusions, 6 – outer borders of shadows. Abbreviations: Arrowheads indicate empodial claws. Abbreviations: *ad –* suckers; ω *–* palp and tarsal solenidia; φ *–* tibial solenidion; *e –* tarsal apical seta; *cox –* coxa; *h –* dorsal setae; *gT –* tibial seta; *hT –* tibial seta; *vF –* femoral setae; *ps –* pseudanal setae; *g –* genital setae; σ *–* genual solenidion. Scale bars: 100 μm

**Diagnosis.***Tritonymph*. Subcapitular remnant large, with 2 pairs of short adoral setae (Fig. 3D). Palps free, long, 2-segmented; palp tarsus with at least 1 solenidion (Figs. 4F; and 5). Dorsal idiosoma sclerotized, punctate; sejugal furrow well developed. Progenital and anal opening well separated. Coxal fields III medially separated, distance between them is distinctly longer than the width of the trochanter III (Fig. 5B–D). Anterior apodemes IV curved in the medial part, distinctly angular. Attachment organ large, with anterior suckers (*ad*_3_) and median suckers (*ad*_1 + 2_) well developed (Figs. 3B; 4D–F; and 5B,–D); conoids *ps*_1_ and *ps*_2_ vestigial. Legs with typical segmentation (trochanter-tarsus). Tarsal empodial claws I-IV present, arising directly from tarsal apices (Fig. 5). Some tarsal setae foliate (Figs. 4F; and 5).

**Remarks**. *Plesioglyphus* belongs to Schizoglyphidae based on the presence of 2 pairs of adoral setae, well-developed palps, 4 pairs of genital setae, transversely elongated cuticular suckers formed by the fusion of pseudanal setae *p*_*1*_ *+ p*_*2*_, and the anal opening situated between suckers *ad*_*1 + 2*_. *Plesioglyphus* gen. n. is very similar to the extant genus *Schizoglyphus*, but differs by the following: the gnathosoma is larger, reaching femora I (distinctly not reaching in *Schizoglyphus*); the distance between suckers *ad*_3_ and *ad*_1 + 2_ is slightly smaller than the diameter of these suckers (slightly larger than the diameter of *ad*_1_ in *Schizoglyphus*); anterior apodemes IV are distinctly curved medially (curved only at tips in *Schizoglyphus*).

**Etymology.** The generic name is formed from two Greek stems, πλησίον (near, neighbouring) and γλῠ́φω (to carve, cut out with a knife, engrave). The former stem is used in the formation of names in paleontology, while the latter stem is widely used to form names in astigmatid mites. Gender masculine.

***Plesioglyphus lebanotermi*** **sp. n.**

urn: lsid: zoobank.org: act: FF680829-45AE-4C88-B568-375C8D2BE18B.

**Description.***Tritonymph*. Gnathosoma large, subcapitular remnant subquadrate (width 1.2 times longer than length) (Fig. 3D). Palps 2-segmented. Palptarsus with at least one a seta and a solenidion ω; solenidion ω subequal or longer than palps (Figs. 3B–C; 4F; and 5). Two pairs short adoral setae present (Fig. 3D). Gnathosoma (except for palps) situated under rostrum.

Rostrum large, wide, rounded, without eyes. Dorsum with propodosomal and hysterosomal shields. Shields roughly punctate, separated by a distinct sejugal furrow. Dorsal setae not observed, except *h*_3_ on posterior hysterosoma.

Ventral side. Sternum present, long, nearly reaching anterior portions of apodemes II (specimen 12). Coxal apodemes II-IV with free ends, anterior apodemes IV distinctly curved medially, angular. Coxal fields I-IV open; coxal fields III well-separated medially, distance between them distinctly longer than width of trochanter III. Coxal setae not observed. Progenital opening nearly as long as base of legs III or IV, situated at level of trochanters IV, well-separated from attachment organ. Genital setae present, at least 4 pairs (Fig. 3B). Genital papillae not observed. Anal opening situated within attachment organ; distance between anal and progenital openings more than twice the length of progenital opening. Attachment organ large, almost as wide as body width. Suckers *ad*_3_ and *ad*_l+2_ large, oval; *ad*_3_ posterior to progenital opening, *ad*_l+2_ lateral to anal opening (Figs. 3B; 4D–F; and 5B–D). Distance between *ad*_3_ and *ad*_l+2_ shorter than diameter of these suckers. Dorsoventral muscles of suckers *ad*_l+2_ well-developed (holotype). Alveolae *ps*_1_ and *ps*_2_ situated between suckers *ad*_3_ and *ad*_l+2_.

Legs short, thick, with typical set of segments. Empodial claws I-IV present, slightly shorter than tarsi (Fig. 5A, B). Tarsus I with seta *e*, long, widened at tip and at least 1–2 other foliate setae, other setae spiniform. Tarsus II similar to tarsus I, but *e* shorter. Tarsi III and IV with at least 6 foliate setae; 1 seta on tarsus IV longer than other (Figs. 4F; and 5C–E). Tibiae I-II with 2 setae (*gT* and *hT*) (Fig. 5,E), setae on tibiae III-IV not observed. Femora I-II with seta *vF* (Fig. 5C). Setation of tibiae III-IV, genua I-IV, femora III-IV and trochanters I-IV not observed. Tarsi I-II with 2 solenidia ω. Tibiae I-II with 1 long solenidion φ (its tip reaches tip of seta *e*) (Figs. 4F; and 5). Tibia III with a single solenidion φ (longer than combined length of genu and tarsus III) (Figs. 3C; and 5). Genu I with one bacilliform solenidion σ (Fig. 5E). Other solenidia not observed.

Measurements (*n* = 3) in micrometers. Idiosoma 182–220 long, 105–110 wide. Prodorsum 68, width 95. Hysterosoma length 115. Gnathosoma 30–35, width 23–25; free palps 14–15, gnathosomal solenidion ω 14–18. Length of attachment organ 50–67, width 66–70, *ad*_3_ 17–22 × 13–19, *ad*_l+2_ 20–28 × 17–24. Legs I: length 46–50, *e* I 24–30, ω I 9–11, φ I 34–36, σ I 10. Legs II: length 37, *e* II 14–22, φ II 20.

**Remarks.** The new species differs from *Schizoglyphus biroi* by long tibial solenidia φ I–II protruding the tips of respective tarsi (not protruding in *S. biroi*). The following 16 phoretic tritonymphs were examined:

Specimen 1 (341 E) (Figs. 2A; 3; and 5C) – holotype, ventral. Legs and most of tarsal setae, gnathosoma (including adoral setae) and muscles are visible; a bubble inside the mite obscures observation.

Specimen 2 (341 F) (Figs. 2A; 4E–F; and 5E) – paratype, dorsolateral. Patterns on the dorsal shields and the posterior part of the attachment organs are well visible; the palp tarsus; legs I, right legs II -IV, anal opening, suckers *ad*_3_ and *ad*_l+2_ are somewhat visible.

Specimen 3 (341 G) (Fig. 2B) – paratype, frontal view. Outlines of the gnathosoma, legs I, and sejugal furrow were observed.

Specimen 4 (341 H) (Figs. 2C; 4D; and 5D) – paratype, ventral. The gnathosoma, suckers *ad*_3_ and *ad*_l+2_, *ps*_1_, anal and genital opening, some coxal apodema, and right tibial solenidion φ II are visible; legs are somewhat visible as outlines.

Specimens 5–11 (341 G–O), 13 (341 Q), 14 (341 R) (Fig. 2E–F) – paratypes, ventral (5, 8), dorsolateral (6), lateral (7, 9, 13, 14), dorsal (10, 11). In specimens 10 and 11, hysterosomal and propodosomal shields (with rostrum), legs I-II, and sejugal furrow are somewhat visible. Other specimens are poorly visible.

Specimen 12 (341 P)(Fig. 4C) – paratype, ventral. Gnathosoma, legs I-II, genital and anal openings, apodemes I-II, attachment organ, legs III-IV are somewhat visible.

Specimen 15 (341 R)(Fig. 4B) – paratype, ventral. The progenital and anal openings, suckers *ad*_l+2_ and left *ad*_3_, coxal areas are well visible. Legs I-III are somewhat visible.

Specimen 16 (341 T) (Figs. 2F; 4A; and 5A) – paratype, dorsal. The dorsal shields, their sculpture, sejugal furrow, legs I-II with empodial claws are well visible. One dorsal seta (probably *h*_3_) is somewhat visible.

## Evidence for a true phoretic association preserved in amber

The termite *Lebanotermes veltzae* was preserved carrying 16 mites, positioned both dorsally (Fig. 1A–B) and ventrally (Fig. 1C). Specimens 1–4 are located at various points on or near the termite’s body, while specimens 5–16 form a larger cluster over its abdominal wing area (Fig. 1B). Although some specimens were dislodged from the host (specimens 1–3, possibly during the process of amber entrapment, other specimens still remain attached to the wing membrane (specimens 4–16). Notably, specimen 16 has its pretarsal claws, equipped with foliate setae, in direct contact with the membrane of the right hindwing’s dorsal side (Figs. 2D, 2F; and 4A). The presence of 16 conspecific phoretic tritonymphs on the termite host, along with evidence of direct mite-host contact, supports the conclusion that this association is neither taphonomic nor random, but represents a true biological relationship.

## Modern schizoglyphids are found in termite associations

**Fig. 6:**
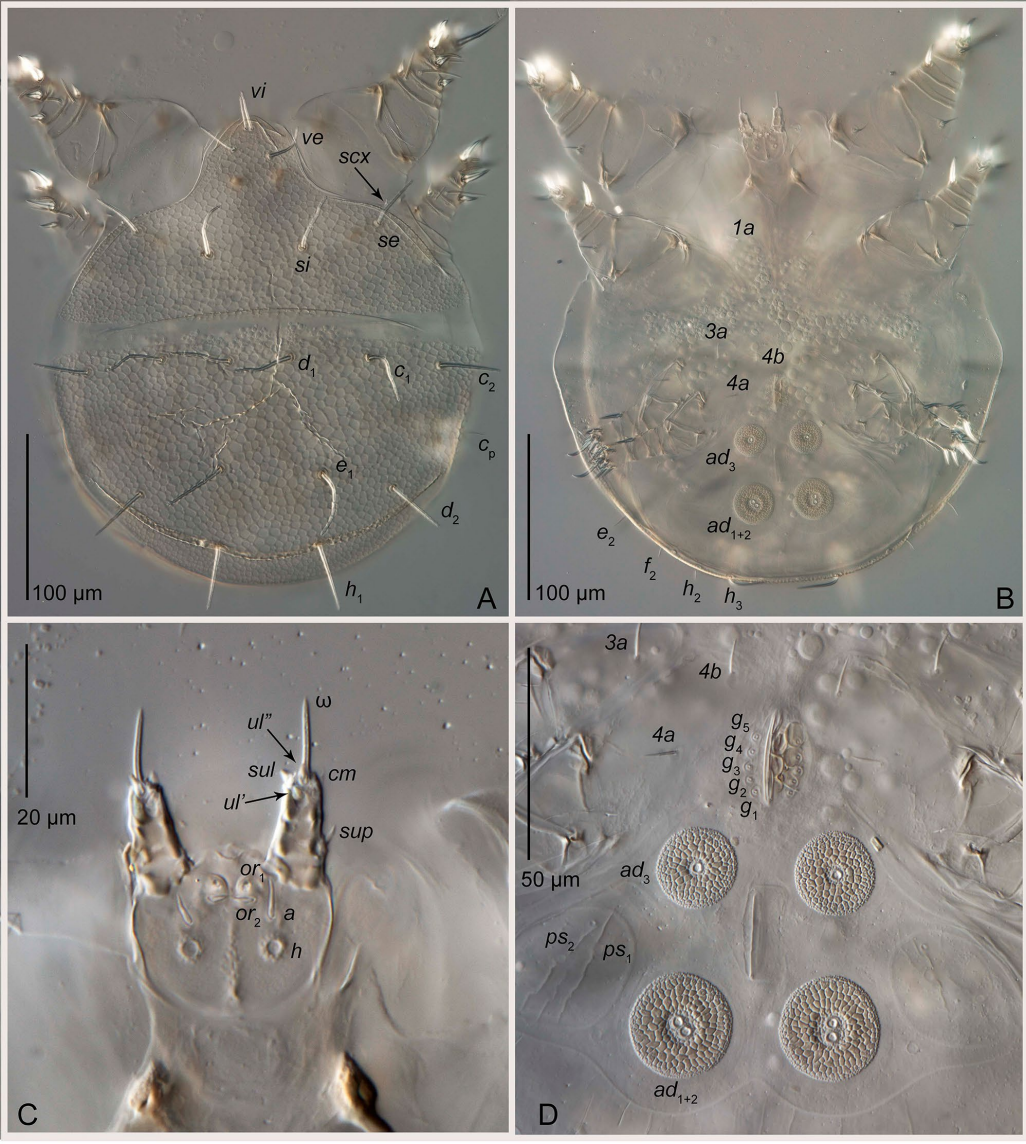
Diagnostic characters of phoretic nymphs of the family Schizoglyphidae as exemplified by a modern species collected from the termite *Stolotermes ruficeps* from New Zealand. (**A**) Dorsal view. (**B**) Ventral view. (**C**) Ventral view of gnathosoma. (**D**) Attachment organ. Abbreviations: *vi*, *ve*, *si*, *se*– prodorsal setae; *scx*– supracoxal seta; *c*, *d*, *e*, *f*– hysterosomal setae; *1a*, *3a*, *4a*, *4b*– coxal setae; *g*– genital setae; *ad–* suckers; *ps–* pseudanal setae; *or*– adoral setae; *h*– hysterosomal and gnathosomal setae; ω *–* palpal solenidion; *sup*, *cm*, *ul*, *sul*– palpal setae. Scale bar 100 μm (**A**, **B**), 20 μm (**C**), 50 μm (**D**)

The earliest known crown-group astigmatid, *Schizoglyphus biroi*, a heteromorphic phoretic tritonymph, was found on the tenebrionid beetle *Dioedus tibialis* (= *Tagalus tibialis*) in western New Guinea [34]. Despite extensive efforts to re-collect these mites from similar hosts (OConnor, pers. comm.), no further specimens were found, suggesting that this record was incidental rather than indicative of a true biological association. Another reported occurrence of “*Schizoglyphus* sp.” on a scarabaeid larva from India [35] actually represents the genus *Sancassania* (family Acaridae). Here, we report the discovery of five tritonymphs of *Schizoglyphus* sp. found in galleries of the New Zealand wetwood termite *Stolotermes ruficeps* (family Stolotermitidae), in a *Pinus radiata* log in Thames, New Zealand (Dickson Holiday Park, Tinker Trail, 37°06’42.7"S 175°31’22.1"E). The termite specimens were ethanol-washed from alate females, workers, and immature termites. These mites display several key character states that unequivocally place them within Schizoglyphidae: three-segmented palps (Fig. 6C), three pairs of genital papillae, five pairs of genital setae (Fig. 6B and D), the gnathosoma bearing adoral setae (Fig. 6B–C), the cuticular suckers of the attachment organ are composed of *p*_*1*_ *+ p*_*2*_ (with alveoli), and the anal opening situated between suckers *ad*_*1 + 2*_ (Fig. 6B and D). These specimens represent a new species, which will be described in a future publication. Additionally, a different species of tritonymphal *Schizoglyphus* sp. was previously collected from an alate queen of *Stolotermes ruficeps* in New Zealand, although these specimens were unfortunately lost (B. OConnor, pers. comm.). These new findings strongly suggest that schizoglyphids live inside termite nests, likely as inquilines, and use founder alate termites for dispersal from one nest to another.

## Discussion

The nests of social insects, such as termites, are stable, long-term habitats that provide abundant food resources, attracting a diverse array of inquiline organisms, including mites, flies, beetles, antlions, wasps, true bugs, silverfish, springtails, woodlice, harvestmen, pseudoscorpions, spiders, millipedes, and gastropods [9, 36–38]. Among these, Astigmata stands out as the most speciose lineage of termitophiles (see also Table 1 in supplementary materials) [39].

Remarkably, despite several records of fossilized astigmatans on alate termites, very few are reported from modern alate termites. We are confident interpreting this observation as an observation bias. First, mites are not easily noticed on alate hosts. Indeed, when mites are phoretic of alate reproducers of social insects the phoront often is hidden underneath the wings, at body contact, as in the bee *Halictus frontalis* [40]. Second, biologists may overlook associations until they are shown in the fossil record, prompting future publication of extant records. For example, springtails were reported as being phoretic on diverse insect hosts in amber, including on alate social insects, with the prediction that these interactions shall still exist [e.g. 38, 42–43]. Modern springtails were since indeed observed from the body of flies, tipulids and alate termites (N.R. pers. obs). Finally, alate termites are not regularly examined for mites because they have a short lifespan compared to other castes of social insects, being produced only at a certain time of year and losing their wings right after nuptial flight. In contrast, alate casts in amber are not uncommon (both males and females), with resin patches forming along the barks mostly above ground level, trapping the flying individuals. This is illustrated by amber termite-mite records: unidentified mites and Mesostigmata were found on extinct Euisoptera [37] from Jordanian amber with a similar Early Barremian age as Lebanese amber [43] (~ 130 Ma) and a Cenomanian (~ 100 Ma) termite-like roach from Burmese amber [44].

Astigmatan heteromorphic nymphs, including *Plesioglyphus lebanotermi*, have a highly specialized attachment organ (Figs. 4, 5 and 6) serving for attachment and hitchhiking on hosts [14, 45]. The success of a heteromorphic nymph attachment depends on its location on the host’s body, to both prevent detachment by the host and minimize interference with the host’s locomotion abilities [46]. Among the 16 individuals of *P. lebanotermi*, most specimens were found on the anteriormost portions of the wings, on areas of overlap between hindwings (sp. 5–15, Fig. 1 and 2D), or between hindwing and forewings (sp. 16, Figs. 1; 2F; and 5E) suggesting that the fossil tritonymphs were attached in the most confined wing areas that were available to their grasp.

As astigmatid heteromorphic nymphs lack a mouth, oral feeding is not possible. Still, feeding as a parasite can occur during phoresy via the attachment organ suckers, anus or genital papillae [1, 47]. Inside the nest, certain mites (such as *Australhypopus* sp.) may provide sanitary functions by feeding on dead termite corpses [5–6]. During phoresy, mites can potentially impede the mobility of their hosts when the mite loads are high [48–49]; but, in general, phoresy is harmless to them. The significant advantage gained by phoronts is linked to an increased dispersal distance, allowing them to exploit new food resources or access different hosts. In social insects, dispersal is enhanced during periods of colony swarming through attachment to alate reproducers. At swarming, alate termites can travel distances of over one kilometer [50]. The observed ~ 130 Ma old association of *Plesioglyphus lebanotermi* with *Lebanotermes veltzae* most likely represents phoretic commensalism, while non-phoretic stages of the mites likely live inside termite nests as commensals.

**Fig. 7:**
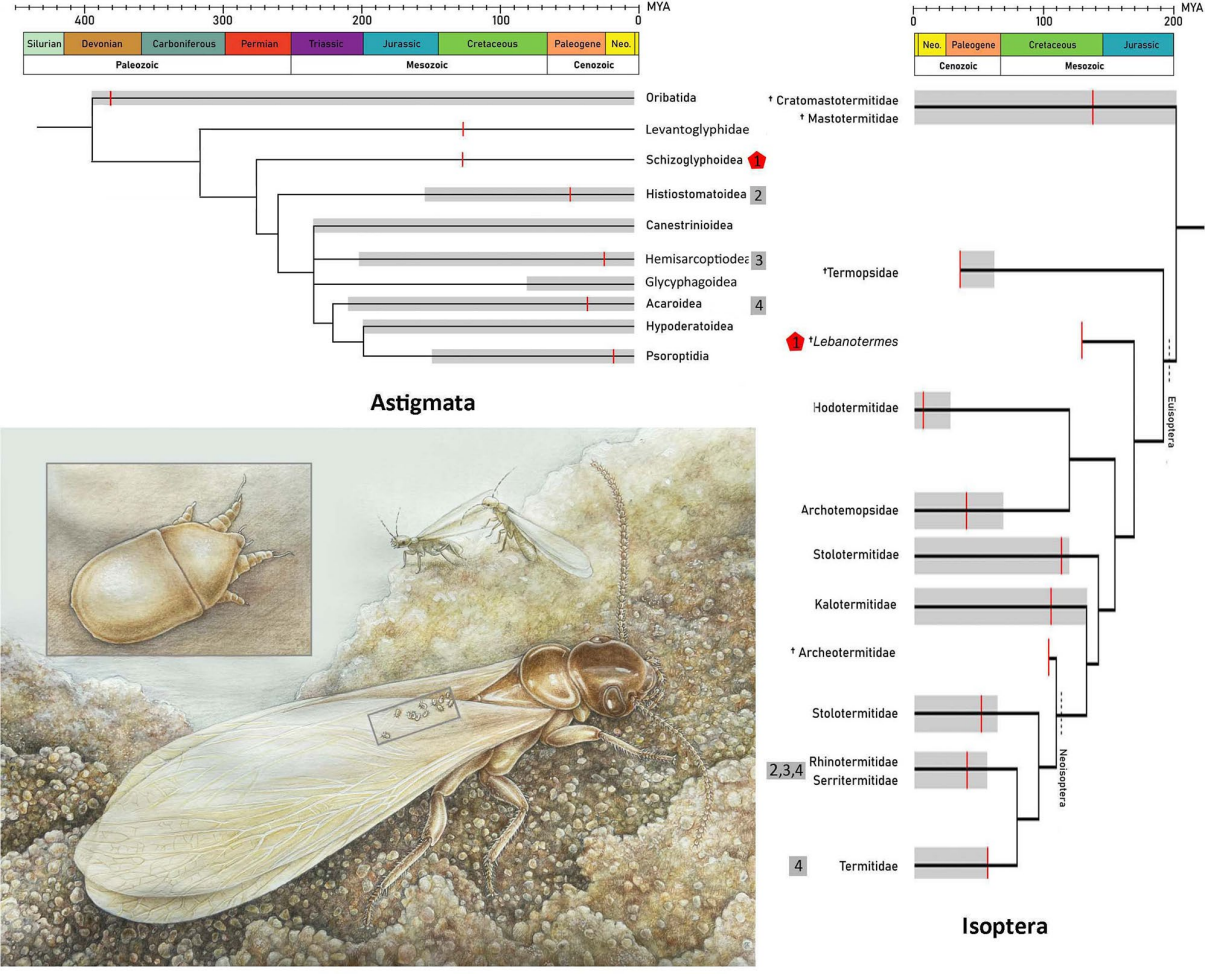
Simplified phylogenies of Astigmata and Isoptera correlated on a geological time scale with marked termite-mite associations and a reconstruction of the described termite-mite association. For a full list of Astigmata/termite associations see Table 1 in supplementary materials. Red dots indicate the herein described mite/termite association and grey boxes the other (extant) associations. Each association is indicated by a number. Grey scale bars reveal occurrence of the clade based on molecular data, while red lines indicate fossil calibrations. Note that the phylogeny of Astigmata is morphologically based and superfamilies appear to be non-monophyletic. Therefore, their origin based on molecular data might differ somewhat in reality. Data for Astigmata phylogeny is used from Seeman & Walter [5] and for Isoptera phylogeny from Jouault et al. [51]. The reconstruction was made by Júlia Káčerová

Four distinct superfamilies of Astigmata are associated with four different termite lineages (Fig. 7; Table 1 in supplementary materials). Among these, the superfamily Acaroidea is the most commonly reported, with 13 genera and 21 species identified from members of the Rhinotermitidae (including *Psammotermes hypostoma*, *Reticulitermes flavipes*, and *Coptotermes formosanus*) and one Termitidae species (*Cornitermes cumulans*) (Fig. 7; Table 1 in supplementary materials). The superfamily Histiostomatoidea has been recorded on several rhinotermitid species, while a species of the superfamily Hemisarcoptoidea has been found on *Psammotermes hypostoma* (Rhinotermitidae). The frequent association of Acaroidea with termites is expected, as this superfamily is species-rich, comprising 562 species [1]. However, despite comparable species diversity (576 species), there are fewer reports of phoretic associations involving Histiostomatidae. Hemisarcoptoidea is a smaller group, with 144 species. All three of these mite superfamilies are associated with Neoisopteran termites, which are believed to have diverged approximately 110 million years ago (with the split between Termopsidae and Rhinotermitidae occurring around 85 Ma [50], Fig. 7).

In contrast, both modern and fossil schizoglyphoid associations, including those reported here, are found on termite lineages that diverged much earlier—from the Early Barremian (126 Ma for Stolotermitidae) to the Late Lias (185 Ma for *Lebanotermes*, Euisoptera; [51]). This distribution may suggest a degree of specialization among phoretic astigmatid mites for specific termite lineages, possibly selected through co-occurrence over geological time and maintained to the present day (Fig. 7). However, the limited number of schizoglyphid occurrences currently prevents any definitive assessment of this pattern.

Schizoglyphid phoretic nymphs retain several plesiomorphic traits when compared to other astigmatid mites. These include: (i) A gnathosomatic remnant with two pairs of adoral setae and relatively long palps, whereas other astigmatids lack adoral setae and have reduced palps; (ii) Three pairs of genital papillae (though this was not observed in *Plesioglyphus lebanotermi*) and four or more pairs of genital setae, while other astigmatids typically have two pairs of genital papillae and only one pair of genital setae; (iii) Pseudanal setae *p*_*1*_ *+ p*_*2*_ are fused into large, transversely elongated cuticular suckers, unlike the small, rounded suckers seen in most astigmatids; (iv) The anal opening is positioned more posteriorly than in most astigmatids, situated at the level of *ad*_*1 + 2*_, rather than *ad*_*3*_ as is typical in other astigmatids. There are interesting similarities between *Plesioglyphus* and the phoretic nymphs of the genus *Levantoglyphus* (family Levantoglyphidae), recently reported from Lebanese amber without host information [13]. Both genera share a well-developed gnathosomal remnant with long palps and long terminal solenidia (ω), indicating the importance of host-seeking behaviour in both lineages. However, *Levantoglyphus* possesses rudimentary chelicerae, enabling food shredding in non-phoretic stages, whereas modern schizoglyphids from New Zealand retain a mouth and pharynx, suggesting that the reduction of functional mouthparts in astigmatid phoretic nymphs was a gradual evolutionary process.

*Plesioglyphus lebanotermi* displays all the synapomorphies of modern schizoglyphid mites and documents the existence of non-feeding, phoretic heteromorphic nymphs in astigmatid mites from the Early Cretaceous (~ 130 Ma). This fossil represents the earliest known crown-group Astigmata with a confirmed phoretic association with termites—a relationship that has persisted into modern times. Its placement among living mites will allow for precise calibration of molecular clock phylogenies. In contrast, the transitional deutonymphs of the extinct stem-group family Levantoglyphidae, whose host associations remain unknown, provide a less precise calibration for molecular clock phylogenies. As these deutonymphs belong to a stem-group lineage of Astigmata, their use in calibrating molecular clocks is limited to a broader and less specific range compared to *Plesioglyphus lebanotermi*.

## Conclusion

The oldest known biotic association of arthropods preserved in amber, dating to approximately 130 million years ago, involves Astigmata, a group of mites specialized in phoresy, which were found attached to a winged termite. This discovery represents the earliest known instance of phoretic mites associated with an arthropod. The mites belong to the genus *Plesioglyphus* n. gen. of the family Schizoglyphidae, an early-diverging lineage of Astigmata currently recognized from a single described species. Remarkably, the plesiomorphic features of these ancient mites —such as long palps and a large subcapitulum— have been highly conserved over 130 million years. In the Early Cretaceous of Lebanon, these schizoglyphids coexisted with other extinct Astigmata (*Levantoglyphus*) that also exhibited plesiomorphic mouthparts. However, unlike these extinct relatives, the schizoglyphids described here are the earliest known crown-group Astigmata to display strictly non-feeding, heteromorphic nymphs. This finding reveals a specialized phoretic relationship with their termite host, suggesting that *Plesioglyphus* functioned as an inquiline in its feeding stages, and dispersed on winged termites via phoresy during non-feeding, heteromorphic stages. This association, which has persisted into modern times, highlights the long-standing evolutionary relationship of Schizoglyphidae with eusocial insects — a connection that has been largely overlooked. Our findings show the remarkable diversity and evolutionary persistence of modern Schizoglyphidae, which continue to exhibit phoresy on termites, reflecting their ancient and ongoing relationship with these insects.

## Electronic supplementary material

Below is the link to the electronic supplementary material.

Supplementary Material 1

## Data Availability

The specimens in this study are deposited in the collection that is mentioned in the Material and Methods section. All other data supporting these findings is included in Table 1 in the supplementary materials.
