# Supplementary material for: The oldest continuous association between astigmatid mites and termites preserved in Cretaceous amber reveals the evolutionary significance of phoresy
Source: BMC Ecol Evol. 2025 Feb 25;25:16. doi: 10.1186/s12862-025-02351-5 (PMC11853500; doi:10.1186/s12862-025-02351-5)
Supplement: Supplementary file 1 — Supplementary Material 1 [file 12862_2025_2351_MOESM1_ESM.docx]

Supplementary Materials

**The oldest continuous association between astigmatid mites and termites preserved in Cretaceous amber reveals the evolutionary significance of phoresy**

Hemen Sendi, Pavel B. Klimov , Vasiliy B. Kolesnikov, Júlia Káčerová, Enrico Bonino, Dany Azar, Ninon Robin

Corresponding authors: [hemen.sendi@savba.sk](mailto:hemen.sendi@savba.sk); danyazar@ul.edu.lb

**The PDF file includes:**

Supplementary Table 1

**Table 1.** Blattodea-mites fossil associations and modern termite-astigmatans associations. We note that cases involving *Australhypopus*, *Acotyledon*, *Caloglyphus,* Rhizoglyphus, *Acarus* and *Calvolia* were likely misidentified to generic or species levels. ^1^ Generalist mite taxa not restricted to one blattodean species. Mites were discovered in all casts, including termite alates.

| **Mite family** | **Host (Blattodea)** | **Association ; attachment location or cast** | **Locality** | **Age** | **References and taxon** |
| --- | --- | --- | --- | --- | --- |
| Undet. | *Ol xiai*  (Olidae) | Unknown; unknown | Myanmar amber | Cenomanian (~ 100 Ma) | Vršanský and Wang, 2017; [1] |
| Undet. | *Bubosa poinari*  (Blattidae) | Syninclusion | Myanmar amber | Cenomanian (~ 100 Ma) | Šmídová, 2020; [2] |
| Undet. | *Facioblatta perfidia* (Liberiblattinidae) | Syninclusion | Myanmar amber | Cenomanian (~ 100 Ma) | Hinkelman, 2022; [3] |
| Undet. | *Magniocula paradoxona*  (Corydiidae) | Likely parasitic; on leg | Myanmar amber | Cenomanian (~ 100 Ma) | Vršanský et al., 2023; [4] |
| Undet. | *Ardatermes hudaludi*  (Euisoptera) | Phoretic; on wings | Jordanian amber | ? Early Barremian (~ 130 Ma) | Kaddumi, 2005; [5] |
| Mesostigmata | *Pabuonqed eulna* (Pabuonqedidae) | Syninclusion & phoretic?; thoracic tergites 2/3 | Myanmar amber | Cenomanian (~ 100 Ma) | Vršanský et al., 2019; [6] |
| Schizoglophidae (Schizoglophoidea) | *Lebanotermes veltzea*  (Euisoptera) | Phoretic; on leg, and wings | Lebanese amber | Early Barremian (~ 130 Ma) | Our data  Plesioglyphus lebanotermi |
| Schizoglophidae (Schizoglophoidea) | *Stolotermes ruficeps* (Stolotermitidae) | Phoretic; unknown (alate) | New Zealand | extant | Barry OConnor, pers. comm. |
| Schizoglophidae (Schizoglophoidea) | *Stolotermes ruficeps*  (Stolotermitidae) | In nests; unknown (all casts) | New Zealand | extant | Our data  undescribed genus |
| Rosensteiniidae (Glycyphagoidea) | *Blaberus gigantea*  (Blaberidae) | Unknown, unknown | Unknown | extant | Haitlinger, 1999; [7]  *Rosensteinia moradorae* |
| Rosensteiniidae (Glycyphagoidea) | *Blaberus gigantea*  (Blaberidae) | Unknown, unknown | Unknown | extant | Haitlinger, 1999; [7]  *Rosensteinia toddy* |
| Rosensteiniidae (Glycyphagoidea) | *Blaberus gigantea*  (Blaberidae) | Unknown, unknown | Unknown | extant | Haitlinger, 1999; [7]  *Rosensteinia rafi* |
| Acaridae (Acaroidea) | *Nauphoeta* sp.  (Blaberidae) | Unknown; unknown | Unknown | extant | Fan and Zahng, 2007; [8]  *Tyrophagus communis* |
| Acaridae (Acaroidea) | *Periplaneta americana*  (Blattidae) | Associated with fungus growth attacking hosts; inside nest | USA | extant | Rau, 1940; [9]  *Rhizoglyphus tarsalis* |
| Acaridae (Acaroidea) | *Psammotermes hypostoma*  (Rhinotermitidae) | Phoretic; inside nest | Egypt | extant | Negm, 2007; [10]  *Caloglyphus manuri* ^1^ |
| Acaridae (Acaroidea) | *Psammotermes hypostoma*  (Rhinotermitidae) | Phoretic; inside nest | Egypt | extant | Eraky, 2000; [11]  *Caloglyphus ornatus*^1^ |
| Acaridae (Acaroidea) | *Psammotermes hypostoma*  (Rhinotermitidae) | Phoretic; inside nest | Egypt | extant | Fakeer et al., 2014; [12]  *Caloglyphus subterraneousi* ^1^ |
| Acaridae (Acaroidea) | *Psammotermes hypostoma*  (Rhinotermitidae) | Phoretic; inside nest | Egypt | extant | Eraky and Osman, 2008; [13]  *Acotyledon ahmadi* ^1^ |
| Acaridae (Acaroidea) | *Psammotermes hypostoma*  (Rhinotermitidae) | Phoretic; inside nest | Egypt | extant | Eraky, 1999; [14]  *Acarus solimani* |
| Acaridae (Acaroidea) | *Reticulitermes flavipes*  (Rhinotermitidae) | Phoretic; mostly on head, less on legs | USA, China | extant | Wang et al., 2002; [15]  *Australhypopus* sp.^1^ |
| Acaridae (Acaroidea) | *Cornitermes cumulans*  (Termitidae) | Phoretic; unknown | Brazil | extant | Pisno et al., 2023; [16]  *Australhypopus* sp.^1^ |
| Acaridae (Acaroidea) | *Coptotermes formosanus*  (Rhinotermitidae) | Phoretic; inside nest | Japan | extant | Phillipsen and Coppel, 1977; [17]  *Australhypopus formosani* ^1^ |
| Acaridae (Acaroidea) | *Psammotermes hypostoma*  (Rhinotermitidae) | Phoretic; inside nest | Egypt | extant | Fakeer et al., 2014; [12]  *Acotyledon termesi* ^1^ |
| Acaridae (Acaroidea) | *Psammotermes hypostoma*  (Rhinotermitidae) | Phoretic; inside nest | Egypt | extant | Eraky, 1998; [18]  *Mahunkaglyphus solimani* |
| Acaridae (Acaroidea) | *Psammotermes hypostoma*  (Rhinotermitidae) | Phoretic; inside nest | Egypt | extant | Eraky, 1998; [18]  *Acotyledon lamiai* ^1^ |
| Acaridae (Acaroidea) | *Psammotermes hypostoma*  (Rhinotermitidae) | Phoretic; inside nest | Egypt | extant | Eraky, 1999; [14]  *Acotyledon longsetoses* ^1^ |
| Acaridae (Acaroidea) | *Psammotermes hypostoma*  (Rhinotermitidae) | Phoretic; inside nest | Egypt | extant | Eraky, 1998; [18]  *Forcellinia egyptiaca* |
| Acaridae (Acaroidea) | *Psammotermes hypostoma*  (Rhinotermitidae) | Phoretic; inside nest | Egypt | extant | Eraky, 1990; [19]  *Forcellinia assiuti* |
| Acaridae (Acaroidea) | *Psammotermes hypostoma*  (Rhinotermitidae) | Phoretic; inside nest | Egypt | extant | Eraky, 1999; [14]  *Cosmoglyphus barbisetus* |
| Acaridae (Acaroidea) | *C. formosanus*  (Rhinotermitidae) | Phoretic, mostly on head | China | extant | Wang et al., 2002; [15]  *Cosmoglyphus absoloni* |
| Acaridae (Acaroidea) | *Psammotermes hypostoma*  (Rhinotermitidae) | Phoretic; inside nest | Egypt | extant | Eraky, 1999; [14]  *Froriepia negmi* |
| Acaridae (Acaroidea) | *Psammotermes hypostoma*  (Rhinotermitidae) | Phoretic; inside nest | Egypt | extant | Eraky, 1998; [18]  *Mahunkallinia serratus* |
| Acaridae (Acaroidea) | Coptotermes sp.  (Rhinotermitidae) | Phoretic; on head | Brazil | extant | Silva et al., 2016; [20]  Rhizoglyphus echinopus |
| Acaridae (Acaroidea) | *Coptotermes formosanus*  (Rhinotermitidae) | Phoretic; unknown | China? | extant | Chen et al., 2022; [21]  *Acarus farris* |
| Acaridae (Acaroidea) | *R. flavipes, R. virginicus, C. formosanus*  (Rhinotermitidae) | Opportunistic; on termite and rearing medium | USA, China | extant | Wang et al., 2002; [15]  *Schwiebea* sp. |
| Acaridae (Acaroidea) | *Pseudacanthotermes spiniger*  (Termitidae) | Phoretic; unknown | Angola | extant | Mahunka, 1963; [22]  *Machadoglyphus termitophilus* |
| Acaridae (Acaroidea, 2 species) | *Pseudacanthotermes spiniger*  (Termitidae) | Phoretic; unknown | Angola | extant | Mahunka, 1963; [22]  *Machadoglyphus minimus* |
| Acaridae (Acaroidea) | *Macrotermes* sp. (Termitidae) | Phoretic; unknown | China | extant | Samšiňák, 1965; [23]  *Schwiebea chinica* |
| Acaridae (Acaroidea) | *Macrotermes* sp. (Termitidae) | Phoretic; unknown | China | extant | Samšiňák, 1965; [23]  *Terglyphus padrtorum* |
| Hemisarcoptidae (Hemisarcoptoidea) | *Psammotermes hypostoma*  (Rhinotermitidae) | Phoretic; inside nest | Egypt | extant | Eraky, 1998; [18]  *Calvolia zaheri* ^1^ |
| Hemisarcoptidae (Hemisarcoptoidea) | *Psammotermes hypostoma*  (Rhinotermitidae) | Phoretic; inside nest | Egypt | extant | Eraky, 1999; [14]  *Calvolia solimani* ^1^ |
| Histiostomatidae (Histiostomatoidea) | *Psammotermes hypostoma*  (Rhinotermitidae) | Phoretic; inside nest | Egypt | extant | Eraky et al., 2010; [24]  *Caloglyphus problematica*  (wrong identification) |
| Histiostomatidae (Histiostomatoidea) | *Coptotermes formosanus*  (Rhinotermitidae) | Phoretic; on legs | USA, China | extant | Wang et al., 2002; Phillipsen and Coppel, 1977; Hughes and Jackson, 1958  ; [15, 17, 25]  *Histiostoma formosana* |
| Histiostomatidae (Histiostomatoidea) | *R. flavipes , R. virginicus , C. formosanus*  (Rhinotermitidae) | Phoretic; mostly on head | USA, China | extant | Wang et al., 2002; [15]  *Histiostoma* sp. |
| Histiostomatidae (Histiostomatoidea) | Blattaria | Phoretic; unknown | Indonesia | extant | Hughes and Jackson, 1958; [25]  *Bonomoia primitiva* |
| Histiostomatidae (Histiostomatoidea) | Blattaria | Unknown, unknown | Indonesia | extant | Hughes and Jackson, 1958; [25]  *Histiostoma banjuwangicum* |
| Histiostomatidae (Histiostomatoidea) | Blattaria | Unknown, unknown | Indonesia | extant | Hughes and Jackson, 1958; [25]  *Histiostoma cirratum* |
| Histiostomatidae (Histiostomatoidea) | Blattaria | Unknown, unknown | Indonesia | extant | Hughes and Jackson, 1958; [25]  *Anoetus indicus* |
| Histiostomatidae (Histiostomatoidea) | Blattaria | Unknown, unknown | Indonesia | extant | Hughes and Jackson, 1958; [25]  *Histiostoma longipes* |
| Histiostomatidae (Histiostomatoidea) | Blattaria | Unknown, unknown | Indonesia | extant | Hughes and Jackson, 1958; [25]  *Lipstorpia mixta* |

**References**

1. Vršanský P, Wang B. A new cockroach, with bipectinate antennae,(Blattaria: Olidae fam. nov.) further highlights the differences between the Burmite and other faunas. Biol. 2017;72(11):1327-33. https://doi.org/10.1515/biolog-2017-0144

2. Šmídová L. Cryptic bark cockroach (Blattinae: *Bubosa poinari* gen. et sp. nov.) from mid-Cretaceous amber of northern Myanmar. Cretac Res 2020;109:104383. <https://doi.org/10.1016/j.cretres.2020.104383>

3. Hinkelman J. Origins and diversity of spot-like aposematic and disruptive colorations among cockroaches. Biol. 2022:1–19. <https://doi.org/10.1007/s11756-022-01163-y>.

4. Vršanský P, Palková H, Vršanská L, Koubová I, Hinkelman J. Mesozoic origin-delayed explosive radiation of the cockroach family Corydiidae Saussure, 1864. Biol. 2023;78:1627–58. <https://doi.org/10.1007/s11756-022-01279-1>

5. Kaddumi HF. Amber of Jordan. The oldest prehistoric insects in fossilised resins. Amman, Jordan; Eternal River Museum of Natural History; 2005.

6. Vršanský P, Koubová I, Vršanská L, Hinkelman J, Kúdela M, Kúdelová T, et al. Early wood-boring ‘mole roach’reveals eusociality “missing ring.” AMBA projekty. 2019;9(1):1–28.

7. Haitlinger R. Three new species of rosensteiniid mites (Acari: Rosensteiniidae: Rosensteiniinae) associates with cockroaches (Blattodea). An. Inst. Biol. UNAM Zool.1999;70(2):79-91.

8. Fan Q-H, Zhang Z-Q. Revision of some species of *Tyrophagus* (Acari: Acaridae) in the Oudemans Collection. Syst Appl Acarol. 2007;12:253–80. <https://doi.org/10.11158/saa.12.3.11>

9. Rau, P. The life history of the American cockroach, *Periplaneta americana* Linn.(Orthop.: Blattidae). Entomol. News. 1940;51(6):151-55.

10. Negm MW. Taxonomy and ecology of some acarid and histiostomatid mite species in Assiut Governorate. PhD Thesis, Faculty of Agriculture, Assuit University, Egypt; 2007.

11. Eraky SA. Identification key for some acaridae mites, hypopi acari: Astigmata with descriptions of two new species. Assiut J Agri Sci. 2000;31(2):341–71

12. Fakeer M, Eraky SA, Ahmed MAI, Desoky ASS. Identification Key for some acarid mites extracted from termite nests with description of two new species. Assiut J Agri Sci. 2014;45(1):68–82.

13. Eraky SA. Osman M. New identification key for some Acaridides (Acaridida) from Upper Egypt, with description of a new Acaridae species. AJESA. 2008;2:49–60. <https://doi.org/10.21608/AJESA.2008.4980>

14. Eraky SA. A new genus and three new species of mites (Acari: Acaridida) phoretic on termites infesting the camphor trees in Aswan, Egypt. Annls Hist -Nat Mus Natl Hung. 1999;91:209–17.

15. Wang C, Powell JE, O’Connor BM. Mites and nematodes associated with three subterranean termite species (Isoptera: Rhinotermitidae). Fla Entomol. 2002;85(3):499–506. [https://doi.org/10.1653/0015-4040(2002)085[0499:MANAWT]2.0.CO;2](https://doi.org/10.1653/0015-4040(2002)085%5b0499:MANAWT%5d2.0.CO;2)

16. Pisno RM, Ferreira DV, Ferla JJ, Serrão JE. Mite–termite interaction: does termite mortality mediate mite density? Insect Soc. 2023;70:243–49. <https://doi.org/10.1007/s00040-023-00913-8>

17. Phillipsen WJ, Coppel HC. *Acotyledon formosani* sp. n. associated with the Formosan subterranean termite, *Coptotermes formosanus* Shiraki (Acarina: Acaridae-Isoptera: Rhinotermitidae). J Kans Entomol Soc. 1977;50(3):399–409.

18. Eraky SA. *Mahunkaglyphus solimani* gen. and sp. n. and three new species (Acari: Astigmata) described from termite nests, western desert, Egypt. Folia ent hung. 1998;59:241–50.

19. Eraky SA. Taxonomy and ecology of some Acarida mites. Ph. D. Thesis, Hungarian Academy of Science, Budapest, Hungary; 1990.

20. Silva, A. F., Pinto, Z. T., Caetano, R. L., Carriço, C., Sato, T. P., Amorim, M., & Gazeta, G. S. Bulb mites *Rhizoglyphus echinopus* (Fumouze and Robin) associated with subterranean termite (Isoptera) in Brazil. EBJT. 2016;9(1):65–68. <https://doi.org/10.12741/ebrasilis.v9i1.553>

21. Chen Y, Zhang L, Zhang S, Liu B, Zeng W, Li Z. The mite *Acarus farris* inducing defensive behaviors and reducing fitness of termite *Coptotermes formosanus*: implications for phoresy as a precursor to parasitism. BMC Ecol Evo. 2022;22(1):1–9. <https://doi.org/10.1186/s12862-022-02036-3>

22. Mahunka S. Neue Anoetiden und Acariden (Acari) aus Angola. Publ Cult Co Diam Angola. 1963:68: 51–66

23. Samšiňák K. Termitophile Milben aus der VR China, 2. Acaridoidea Reichenbachia. 1965: 5: 291–294.

24. Eraky SA, Abdel-Galil FA, K Bohibah MK. Identification key for Some Phoretic Acarididies (Acari: Acaridida) from Upper Egypt with description of two new species. Assiut J Agri Sci. 2010;41(3):76–92. <https://doi.org/10.21608/AJAS.2010.268136>

25. Hughes RD, CG Jackson. A review of the Anoetidae (Acari). VJS. 1958:5-198
